# Body mass index as a prognostic factor in patients with extranodal natural killer/T-cell lymphoma, nasal type

**DOI:** 10.18632/oncotarget.11373

**Published:** 2016-08-18

**Authors:** Jie Liu, Yao-Tiao Deng, Li Zhang, Na Li, Ming Jiang, Li-Qun Zou, Yu Jiang

**Affiliations:** ^1^ Department of Medical Oncology, Cancer Center, State Key Laboratory of Biotherapy, West China Hospital, Sichuan University, Chengdu, China

**Keywords:** extranodal natural killer/T-cell lymphoma, body mass index, prognosis, radiotherapy, chemotherapy

## Abstract

Epidemiological evidence has shown that body mass index (BMI) can predict survival in several types of cancer. However, the role of BMI in extranodal natural killer/T-cell lymphoma, nasal type (ENKTL) is still unclear. This retrospective single-center study included 251 newly diagnosed patients to determine the prognostic value of BMI in ENKTL. Of these, 203 patients received chemoradiotherapy, 37 received chemotherapy alone, 8 received radiotherapy alone, and 3 received only best supportive care. With a median follow-up of 28 months, the estimated 3-year overall survival (OS) and progression-free survival (PFS) rates were 64.4% and 60.9%, respectively. The receiver-operating characteristic curve showed that 20.8 kg/m^2^ was the optimal cut-off of BMI to predict survival. BMI < 20.8 kg/m^2^ was associated with lower 3-year OS (52.8% *vs.* 72.9%, *P* = 0.001) and PFS (48.8% *vs.* 69.8%, *P* < 0.001) rates. Multivariate analysis indicated that BMI, performance status, lactate dehydrogenase (LDH) levels, chemotherapy, and radiotherapy were independent prognostic factors for OS. Furthermore, BMI, number of extranodal sites, performance status, LDH, and radiotherapy were predictive of PFS. These results suggest that BMI at the cut-off of 20.8 kg/m^2^ might be a prognostic factor in patients with ENKTL.

## INTRODUCTION

Extranodal natural killer/T-cell lymphoma, nasal type (ENKTL) is a rare subset of non-Hodgkin's lymphoma (NHL). It is more frequent in Asia and Latin America, accounting for 7%-10% of all NHLs in these areas, but only 1% in western countries [1]. ENKTL can be dichotomized as nasal disease primarily localized to the upper aerodigestive tract (UAT), or extranasal disease occurring in non-UAT sites (e.g., skin, intestine, testicles) [2]. According to the data from the International Peripheral T-Cell Lymphoma Project, the median overall survival (OS) was only 0.36 and 1.6 years for extranasal and nasal disease, respectively [2]. In 2006, Lee et al. established a prognostic model for ENKTL, known as the Korean Prognostic Index, which contained four factors: B symptoms, serum lactate dehydrogenase (LDH) level, stage, and regional lymph node (RLN) involvement [3]. In this study, most patients (202/262) had received anthracycline-based chemotherapy [3]. Recently, the survival of ENKTL patients has improved owing to early radiotherapy and new chemotherapy regimens containing asparaginase and other non-anthracycline drugs [2, 4-10]. The prognostic index of natural killer lymphoma (PINK) model published in 2016 was developed for patients who received non-anthracycline-based chemotherapy with or without radiotherapy, based on their clinical characteristics including age, stage, distant lymph node (DLN) involvement, and non-nasal type disease [11].

Body mass index (BMI) is one of the common criteria to evaluate the degree of obesity. Increased BMI is associated with a higher risk of diabetes mellitus, cardiovascular diseases [12], as well as cancers [13]. Recent evidence has also shown that abnormal BMI can predict prognosis in many types of cancers [14-23], such as breast [14], colon [15] and liver [22] cancers, and diffuse large B-cell lymphoma (DLBCL) [17-19]. Although there is sufficient evidence in these cancers, the role of BMI in ENKTL is still unclear. Therefore, we carried out this retrospective study to determine the prognostic value of BMI in newly diagnosed patients with ENKTL.

## RESULTS

### Patient characteristics

In total, 301 patients met the inclusion criteria. Fifty cases were excluded. Of these, 28 patients had primary extranasal diseases, 9 were younger than 18 years, 6 were complicated by other types of cancer, and the staging was unclear in 7 patients. The median age of the remaining 251 eligible patients was 42 years (range, 18-86 years), and 170 (67.7%) patients were male. At diagnosis, 40 patients (15.9%) were classified as underweight, 130 (51.8%) as normal weight, 49 (19.6%) as overweight, and 32 (12.7%) as obese according to the Asian criteria of BMI classification. Most (*n* = 203, 80.9%) patients received chemoradiotherapy, 37 (14.7%) received chemotherapy alone, 8 (3.2%) received radiotherapy alone, and 3 (1.2%) received only best supportive care. The chemotherapy regimens included VDLP (etoposide, dexamethasone, L-asparaginase, and cisplatin: 184 patients), LVP (L-asparaginase, vincristine, and prednisone: 36 patients), asparaginase combined with non-anthracycline drugs (e.g., gemcitabine, irinotecan, or dexamethasone: 8 patients), CHOP (doxorubicin, cyclophosphamide, vincristine, and prednisone: 8 patients), and other regimens in 4 patients. Of patients treated with radiotherapy, 205 (97.2%) completed the planned dose (50-56 Gy). By December 2015, 79 patients had died. The median follow-up time was 28 months (range, 8-86 months) for patients who were alive. The estimated 3-year OS and PFS rates were 64.4% and 60.9%, respectively. Patient characteristics are listed in Table 1.

**Table 1:** Patient characteristics

Characteristic	No. of patients	%	Characteristic	No. of patients	%
Gender			Ann Arbor Stage		
Female	81	32.3%	I–II	213	84.9%
Male	170	67.7%	III–IV	38	15.1%
Age			Local invasiveness		
≤60	219	87.3%	No	178	70.9%
>60	32	12.7%	Yes	73	29.1%
B symptoms			RLN involvement		
No	122	48.6%	No	164	65.3%
Yes	129	51.4%	Yes	87	34.7%
Performance status			DLN involvement		
0-1	223	88.8%	No	236	94.0%
2-4	28	11.2%	Yes	15	6.0%
LDH			No. of extranodal sites		
Normal	151	60.2%	1 site	216	86.1%
Elevated	100	39.8%	>1 site	35	13.9%
BMI			Radiotherapy		
<18.5	40	15.9%	No	40	15.9%
18.5-22.9	130	51.8%	Yes	211	84.1%
23-24.9	49	19.6%	Chemotherapy		
≥25	32	12.7%	With asparaginase	228	90.8%
			Without asparaginase	12	4.8%
			No	11	4.4%

### The prognostic value of BMI in patients with ENKTL

**Figure 1:**
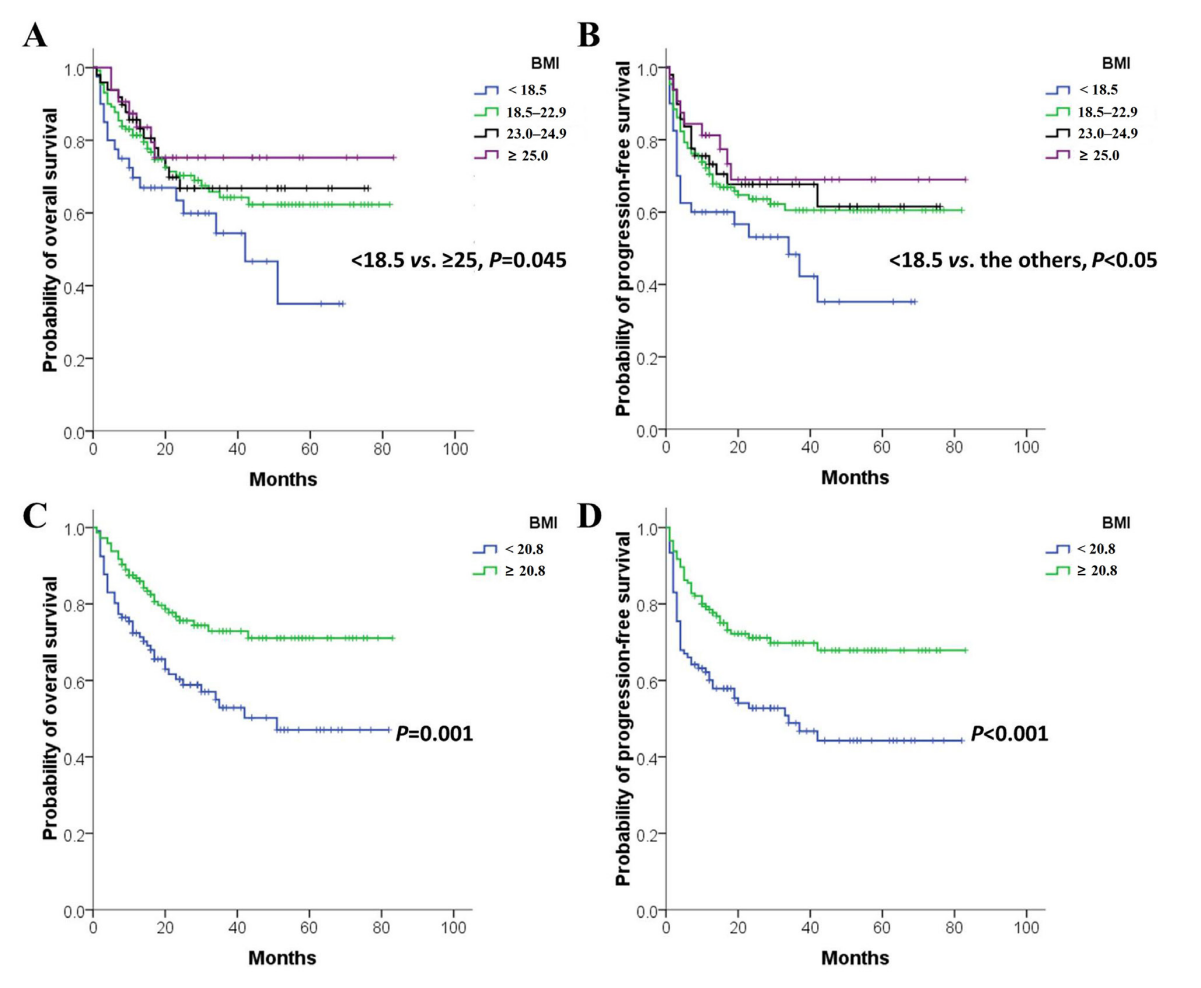
Survival curves of 251 patients diagnosed with ENKTL **A.** and **B.** Patients were stratified into BMI groups according to the Asian criteria: underweight (< 18.5 kg/m^2^), normal weight (18.5-22.9 kg/m^2^), overweight (23.0-24.9 kg/m^2^) and obese (≥ 25.0 kg/m^2^). **C.** and **D.** Patients were divided by the optimal cut-off of BMI (20.8 kg/m^2^).

It was found that the estimated 3-year OS rates were 54.5%, 64.3%, 66.8%, and 75.2%, and the 3-year PFS rates were 48.3%, 60.5%, 66.8%, and 69.0% for underweight, normal weight, overweight, and obese patients, respectively. Survival curves showed that the significant difference of OS was only between underweight and obese patients (*P* = 0.045, Figure 1A). The PFS of the underweight group was shorter than that of other groups (all *P* < 0.05), and similar PFS curves were observed in normal weight, overweight and obese patients (Figure 1B). Therefore, patients were dichotomized into the following groups: BMI < 18.5 kg/m^2^ and BMI ≥ 18.5 kg/m^2^. Patients with BMI < 18.5kg/m^2^ had inferior 3-year OS (54.5% *vs*. 66.3%, *P* = 0.035) and PFS (48.3% *vs*. 63.4%, *P* = 0.017) rates than those with BMI ≥ 18.5 kg/m^2^. However, BMI at the cut-off of 18.5 kg/m^2^ was not an independent prognostic factor for either OS [(hazard ratio (*HR*) = 1.555, 95% confidence interval (*CI*) = 0.903-2.681, *P* = 0.111)] or PFS (*HR* = 1.600, 95% *CI* = 0.955-2.681, *P* = 0.074).

The receiver operating characteristic (ROC) curve analysis indicated that 20.8 kg/m^2^ was the optimal cut-off value of BMI to predict survival (area under the curve = 0.592, *P* = 0.020). Patients with BMI < 20.8 kg/m^2^ had lower 3-year OS (52.8% *vs*. 72.9%, *P* = 0.001, Figure 1C) and PFS (48.8% *vs*. 69.8%, *P* < 0.001, Figure 1D) rates than patients with BMI ≥ 20.8 kg/m^2^. Multivariate analysis indicated that BMI at this cut-off was an independent prognostic factor for both OS and PFS (Table 2). Patients were further stratified to perform subgroup analyses to identify those who might be compromised by a low BMI (BMI < 20.8 kg/m^2^) according to the stage (early stage *vs*. advanced stage), use of radiotherapy (yes *vs*. no), and the regimen of chemotherapy (asparaginase-containing *vs*. non-asparaginase-containing). It was found that in patients with early stage (*n* = 213), receiving radiotherapy (*n* = 211), and undergoing asparaginase-containing chemotherapy (*n* = 228), BMI < 20.8 kg/m^2^ was associated with poor survival outcomes (all *P* < 0.050).

**Table 2:** Multivariate analysis in 251 patients with ENKTL

	Overall survival		Progression-free survival	
Characteristic	*HR (95% CI)*	*P* value	*HR (95% CI)*	*P* value
Gender (male *vs*. female)	1.708 (0.997-2.927)	0.051	1.338 (0.821-2.182)	0.243
Ann Arbor Stage (III–IV *vs*. I–II)	0.000 (0.000- 6.493×10^48^)	0.887	3.114 (0.772- 12.565)	0.111
Local invasiveness (yes *vs*. no)	1.367 (0.790- 2.366)	0.264	1.251 (0.761- 2.057)	0.377
RLN involvement (yes *vs*. no)	1.491 (0.886- 2.510)	0.132	1.514 (0.937- 2.444)	0.090
DLN involvement (yes *vs*. no)	0.970 (0.375- 2.505)	0.949	1.130 (0.457- 2.792)	0.791
No. of extranodal sites (>1 vs. 1)	4534.994 (0.000- 1.913×10)	0.892	6.623 (1.667-26.316)	0.007
B symptoms (yes *vs*. no)	0.832 (0.486-1.426)	0.504	0.841 (0.508- 1.393)	0.501
Performance status (2–4 *vs*. 0–1)	4.172 (2.086- 8.346)	<0.001	2.723 (1.385- 5.354)	0.004
LDH (elevated *vs*. normal)	2.349 (1.390- 3.970)	0.001	1.725 (1.063- 2.797)	0.027
Chemotherapy (per 1-degree increase)^a^	0.612 (0.407-0.922)	0.019	0.717 (0.490- 1.048)	0.086
Radiotherapy (yes *vs*. no)	0.283 (0.149-0.538)	<0.001	0.156 (0.077- 0.316)	<0.001
BMI (<20.8 *vs*. ≥20.8)	1.689 (1.049-2.717)	0.031	1.656 (1.059-2.591)	0.027

Note:

a Patients treated without chemotherapy, with non-asparaginase-based chemotherapy, and with asparaginase-containing chemotherapy were assigned the values 1, 2, and 3, respectively.

### Association between BMI and other characteristics

A greater proportion of patients with BMI < 20.8 kg/m^2^ were female (39.6% *vs*. 26.9%, *P* = 0.033), presented with advanced stage disease (21.7% *vs*. 10.3%, *P* = 0.013), RLN involvement (42.5% *vs*. 29.0%, *P* = 0.027), DLN involvement (11.3% *vs*. 2.1%, *P* = 0.002), and extranodal sites > 1 (19.8% *vs*. 9.7%, *P* = 0.022), and were treated without radiotherapy in first-line treatment (24.5% *vs*. 9.7%, *P* = 0.001) than those with BMI ≥ 20.8 kg/m^2^ (Table 3). In patients receiving antitumor treatment, patients with BMI < 20.8 kg/m^2^ were more likely to receive radiotherapy doses of less than 50 Gy (6.3% *vs*. 0.8%, *P* = 0.030) and chemotherapy of less than 3 cycles (32.0% *vs*. 16.4%, *P* = 0.005) than those with BMI ≥ 20.8 kg/m^2^ (Table 3).

**Table 3:** Association between BMI and other characteristics

Characteristic	BMI <20.8	BMI≥20.8	*P* value
Gender			0.033
Female	42	39	
Male	64	106	
Age			0.844
≤60	93	126	
>60	13	19	
Ann Arbor			0.013
I–II	83	130	
III–IV	23	15	
Local invasiveness			0.742
No	74	104	
Yes	32	41	
RLN involvement			0.027
No	61	103	
Yes	45	42	
DLN involvement			0.002
No	94	142	
Yes	12	3	
Extranodal involvement			0.022
1 site	85	131	
>1 site	21	14	
B symptoms			0.519
No	49	73	
Yes	57	72	
Performance status			0.090
0-1	90	133	
2-4	16	12	
LDH			0.077
Normal	57	94	
Elevated	49	51	
Chemotherapy			0.535^a^
No	6	5	
Yes	100	140	
Chemotherapy cycles			0.005
<3	32	23	
≥3	68	117	
Radiotherapy			0.001
No	26	14	
Yes	80	131	
Radiotherapy dosage			0.030a
<50Gy	5	1	
≥50Gy	75	130	

Note:

a Fisher's exact test.

### Treatment-related adverse events based on BMI

Among 248 patients receiving antitumor treatment, grade 3-4 hematological and hepatic toxicities occurred in 86 (34.7%) and 12 (4.8%) patients, respectively. There was no grade 3-4 nephritic toxicity. Treatment-related mortality rate was 2.4%. Grade 3-4 neutropenia (33.3% *vs*. 19.6%, *P* = 0.014) and treatment interruption (9.5% *vs*. 2.1%, *P* = 0.010) were more likely to occur in patients with BMI < 20.8 kg/m^2^ than in those with BMI ≥ 20.8 kg/m^2^ (Table 4).

**Table 4:** Treatment-related adverse events based on BMI

Adverse event	BMI <20.8	BMI ≥20.8	*P* value
Anemia			0.095
Grade 0-2	93	135	
Grade 3-4	12	8	
Leukopenia			0.055
Grade 0-2	70	111	
Grade 3-4	35	32	
Neutropenia			0.014
Grade 0-2	70	115	
Grade 3-4	35	28	
Thrombopenia			0.154
Grade 0-2	95	136	
Grade 3-4	10	7	
Elevated transaminase			0.289^a^
Grade 0-2	100	140	
Grade 3-4	5	3	
Elevated bilirubin			0.999^a^
Grade 0-2	101	138	
Grade 3-4	4	5	
Elevated creatinine			0.999^a^
Grade 0	103	139	
Grade 1-2	2	4	
Treatment interruption			0.010
No	95	140	
Yes	10	3	
Treatment-related death			0.245^a^
No	101	141	
Yes	4	2	

Note:

a Fisher's exact test.

## DISCUSSION

In this study, we found that BMI at diagnosis was an independent prognostic factor in newly diagnosed ENKTL patients, after being adjusted for other theoretical confounding factors. To the best of our knowledge, our study represents the first large cohort to evaluate the prognostic value of BMI in patients with ENKTL.

The survival rates of ENKTL patients seemed greater with higher BMI, but a statistically significant difference did not exist between normal weight and overweight/obese patients (Figure 1A and 1B). In fact, the prognostic value of overweight/obese status in cancer remains controversial. Obesity, rather than overweight, was associated with higher mortality in breast and colorectal cancers [14, 24], but being overweight or obese improved clinical outcomes of liver, and head and neck cancers [16, 22]. In DLBCL, overweight/obesity was a favorable prognostic factor in two studies conducted in America and Austria [17, 18], but the OS did not seem different among normal weight, overweight, and obese patients in another study by a Korean group [19], which seemed consistent with our findings in ENKTL (Figure 1A).

As similar prognosis were observed in normal weight and overweight/obese patients (Figure 1A and 1B), we adopted a dichotomized classification of BMI with a cut-off of 18.5 kg/m^2^. The survival of patients with BMI < 18.5 kg/m^2^ was poorer than that of patients with BMI ≥ 18.5 kg/m^2^ in ENKTL. It was consistent with previous findings in breast, colon, and head and neck cancers [14, 15, 20, 23]. However, multivariate analysis suggested that BMI at the cut-off of 18.5 kg/m^2^ was not an independent prognostic factor in ENKTL. Therefore, an ROC curve analysis was conducted, which suggested that 20.8 kg/m^2^ was the optimal cut-off value. BMI < 20.8 kg/m^2^ was associated with lower 3-year OS (Figure 1C) and PFS (Figure 1D) rates. Multivariate analysis also indicated that BMI at this cut-off was an independent prognostic factor (Table 2).

A series of studies support that patients with ENKTL can benefit from asparaginase-containing chemotherapy [7-10, 25]. Early or upfront radiotherapy is also an effective treatment to improve clinical outcome of localized disease [2, 4-6]. We confirmed these findings in the present study (Table 2). Moreover, based on the results of multivariate analysis and subgroup analysis, our findings further suggested that BMI could predict the clinical outcome of ENKTL patients who received the current mainstream treatment strategies.

The reason for poor prognosis among low-weight cancer patients is not well known. One explanation is that underweight patients may be more likely to suffer from comorbidities that increase mortality risk, such as higher rate of second malignancies [15]. In our study, 6 patients who were complicated by other types of cancer were excluded. Therefore, it might not be the reason in ENKTL. Another explanation is that low-weight patients might experience chronic undernutrition, which could weaken the immune system [14, 26]. In our study, BMI < 20.8 kg/m^2^ was associated with other adverse prognostic factors and the absence of early radiotherapy (Table 3). Moreover, patients with lower BMI were more likely to receive less radiotherapy doses and chemotherapy cycles in first-line treatment (Table 3) and experience severe neutropenia and adverse event-related treatment interruption (Table 4). These associations might be the reason for poor prognosis among low-weight patients with ENKTL.

There are several potential limitations in this single-institution retrospective study. Firstly, we excluded patients with primarily extranasal disease. Secondly, most patients (184 of 251) were treated with the chemotherapy regimen of VDLP. These factors limit the ability to extrapolate our findings to the entire ENKTL patient population, especially considering that a standardized chemotherapy regimen for ENKTL is not well established. Thirdly, BMI has limitations in evaluating the patients with sarcopenic or non-sarcopenic obesity [27]. Sarcopenia and visceral obesity were associated with poor survival in some malignant tumors [28-30]. Finally, we could not rule out the possibility of other confounding factors, such as socioeconomic status that might influence the BMI of patients.

In summary, our results indicated that BMI was a prognostic factor for patients with ENKTL in the context of current treatment strategy. Prospective multicentric studies are needed to further confirm this finding in the entire ENKTL patient population.

## MATERIALS AND METHODS

### Patient population and data collection

The study was approved by the Ethics Committee of West China Hospital of Sichuan University. We retrospectively analyzed the data of patients diagnosed with ENKTL between July 2008 and April 2015 in our center. Patients were included if: (1) they were newly diagnosed with ENKTL, and (2) their BMI data were available. Patients were excluded if: (1) their diseases primarily occurred in extranasal sites, (2) they were younger than 18 years, (3) they were athletes, (4) they were pregnant or lactating women, (5) they had other types of cancer, and (6) their staging was unclear. Patient characteristics including age, sex, weight, height, Ann Arbor stage, local invasiveness [31], RLN involvement [3], DLN involvement [11], number of extranodal sites, B symptoms, Eastern Cooperative Oncology Group performance status, serum LDH level, first-line treatment regimens, and survival status were recorded. BMI was calculated as weight in kilograms divided by height in meters squared (kg/m^2^). Patients were stratified into BMI groups according to the Asian criteria: underweight (< 18.5 kg/m^2^), normal weight (18.5-22.9 kg/m^2^), overweight (23.0-24.9 kg/m^2^) and obese (≥ 25.0 kg/m^2^) [19]. Progression-free survival was defined as the time interval from diagnosis to disease progression, relapse or death as a result of any cause. Overall survival was defined as the time from diagnosis to death as a result of any cause.

### Statistical analysis

Categorical variables were summarized as frequency counts and were analyzed using the Chi-squared test. If appropriate, Fisher's exact test was performed. The optimal cut-off value of BMI to predict prognosis was calculated using an ROC curve analysis referring to death of patients. Both OS and PFS were estimated using the Kaplan-Meier method, and survival curves were compared using the log-rank test. Only those factors that were statistically associated with survival at the 0.1 level (*P* ≤ 0.100) in the log-rank test were included in the multivariate analysis of Cox proportional hazard model. All statistical tests were performed using SPSS v17.0 (SPSS, Inc., Chicago, IL), and two-sided *P* ≤ 0.05 was considered statistically significant.
